# Comparison of cognitive functioning as measured by the Ruff Figural Fluency Test and the CogState computerized battery within the LifeLines Cohort Study

**DOI:** 10.1186/s40359-017-0185-0

**Published:** 2017-05-12

**Authors:** Jisca S. Kuiper, Richard C. Oude Voshaar, Floor E. A. Verhoeven, Sytse U. Zuidema, Nynke Smidt

**Affiliations:** 1 Department of Epidemiology, University of Groningen, University Medical Center Groningen, Groningen, 9700 The Netherlands; 2 Department of Psychiatry, University of Groningen, University Medical Center Groningen, Groningen, 9700 The Netherlands; 3 University of Groningen, University Medical Center Groningen, University Center for Psychiatry, Groningen, 9700 The Netherlands; 4 Department of General Practice and Elderly Care Medicine, University of Groningen, University Medical Center Groningen, Groningen, 9700 The Netherlands; 5 Department of Geriatrics, University Medical Center Groningen, Groningen, 9700 The Netherlands

**Keywords:** Cognition, Assessment, Ruff Figural Fluency Test, CogState, Executive functions, Neuropsychological tests

## Abstract

**Background:**

The Ruff Figural Fluency Test (RFFT; a pencil and paper test) and the CogState (a computerized cognitive test battery) are well-validated and suitable tests to evaluate cognitive functioning in large observational studies at the population level. The LifeLines Cohort Study includes the RFFT as baseline measurement and incorporated the CogState as replacement for the RFFT at follow-up. It is unknown how these two tests relate to each other. Therefore, the aim of this study is to examine the correlation between the RFFT and the CogState and the impact of demographic characteristics on this association.

**Methods:**

A subcohort of the LifeLines Cohort Study, a large population based cohort study, participated in this study. Correlations between the RFFT and six subtasks of the CogState were examined. Subgroup analyses were performed to investigate the influence of age, education, and gender on the results. With sensitivity analyses we investigated the influence of computer experience and (physical) impairments.

**Results:**

A total of 509 participants (mean age (SD): 53 years (14.6); range 18–87 years) participated in this study. All correlations between the RFFT and the CogState were statistically significant (except for the correlation between the RFFT error ratio and the CogState One Back Task), ranging from -0.39 to 0.28. Stratifying the analyses for age, education, and gender did not substantially affect our conclusions. Sensitivity analyses showed no substantial influence of level of computer experience or (physical) impairments.

**Conclusions:**

Correlations found in the present study were only weak to moderate, indicating that cognitive functioning measured by the RFFT does not measure the same components of cognitive functioning as six subtasks of the CogState. Computerized testing such as the CogState may be very well suited for large cohort studies to assess cognitive functioning in the general population and to identify cognitive changes as early as possible, as it is a less time- and labor intensive tool.

## Background

Dementia is considered a major public health concern because of high prevalence rates and high economic and social burden [1]. Since therapeutic interventions may be most effective in the preclinical stages of dementia, early detection of cognitive impairments is important [2, 3]. Currently, the clinical diagnosis of cognitive impairment or dementia is based on labor-intensive, time-consuming, and therefore very costly paper and pencil neuropsychological testing [4]. In research settings, assessment of cognitive functioning in the population may provide important contributions in identifying risk factors associated with cognitive impairments. Various cognitive tests are available to measure (changes in) cognition in the general population. The LifeLines Cohort Study is a large observational population-based cohort study (*n* = 167,729) in the north of the Netherlands with the overall aim to gain insight into the etiology of healthy ageing [5]. The Ruff Figural Fluency Test (RFFT) is administered in the LifeLines Cohort Study and includes a baseline measurement of cognitive function. The RFFT is a paper and pencil test used to evaluate nonverbal fluency and executive functioning [6–8]. Nonverbal fluency refers to the ability to utilize one or more strategies to generate nonverbal responses to a specific instruction, within limited time, while avoiding response repetition [7, 9]. Executive functions encompass a variety of higher-order cognitive processes, including planning, inhibition, cognitive flexibility, decision-making and self-monitoring [9]. Impairments in executive functioning may have negative effects on people’s everyday life activities, such as the ability to work and attend school, function independently at home, or develop and maintain appropriate social relations [10]. The popularity of including figural fluency tests in cognitive and neuropsychological test batteries has increased in recent years. Particularly the assessment of executive functioning among older adults has received increased interest [11, 12]. A key reason for this is because often one of the first changes in cognitive functioning occur in the domain of executive function [9, 12]. The RFFT is shown to be sensitive to cerebral dysfunction, particularly in the right frontal lobe [7]. Furthermore, the RFFT is sensitive to early changes in cognitive function, present in young and middle-aged persons, which is valuable in large observational studies into the mechanisms of cognitive decline and dementia and it has demonstrated good test-retest reliability and inter-rater reliability [7]. For these reasons, the RFFT has been administered in the baseline assessment of the LifeLines Cohort Study. However, paper and pencil neuropsychological testing is generally labor-intensive, time-consuming and associated with practice effects [13]. Within the LifeLines Cohort Study, particularly scoring of the RFFT was experienced to be time- consuming and therefore costly. In addition, information on different cognitive domains was deemed valuable. Therefore, an alternative cognitive functioning measurement was incorporated in the follow-up measurements of the LifeLines Cohort Study, as replacement of the RFFT. This alternative is the CogState which is a computerized test battery.

Computerized cognitive testing is increasingly used for the detection of cognitive decline [14] and may be uniquely suited as a screening tool in large studies on (change in) cognitive functioning. Compared to standard neuropsychological tests, computerized testing can have important advantages as it might be more sensitive across a wider range of cognitive functioning (less floor and ceiling effects), have more precise recording of responses, and have less test-retest effects [14, 15]. The CogState computerized cognitive battery was included in the LifeLines Cohort Study because it measures multiple domains of cognitive functioning and it is brief, using automated data processing and scoring. It is suitable for research among people from the general population with a wide range of ages and educational levels [15, 16]. Furthermore, the CogState battery has shown to have good test-retest reliability [17] and validity [18, 19].

Within the LifeLines Cohort Study, the CogState Brief Battery is administered. The CogState Brief Battery is specifically developed to monitor cognitive change. It requires little time for administration, and it has shown to have good validity and good sensitivity to changes in cognitive function [16]. The CogState Brief Battery measures attention/vigilance, processing speed, memory, and working memory functions [16]. For this study, we also included a measurement for executive functioning in order to compare results on executive function as measured by the CogState and the RFFT. Although the CogState offers multiple tests on executive functioning, one specific test on executive functioning for this study (i.e. Groton Maze Learning Test) was chosen in order to minimize the time required to finalize the battery. We chose the Groton Maze Learning Test because it corresponds most with functions that are also needed to perform the RFFT (i.e. nonverbal fluency; the ability to utilize one or more strategies to generate non-verbal responses to a specific instruction, within limited time, while avoiding response repetition). Whereas the other executive functioning tasks of the CogState rely more specifically on inhibition or set shifting.

Although both RFFT scores and CogState scores have been compared to other cognitive tests on various cognitive domains, there is no study that directly compared these cognition tests with each other. Furthermore, most studies investigating the performance of the CogState or RFFT were conducted in a clinical research setting [16, 18, 20, 21], whereas only few studies were conducted in the general population including individuals of all ages and educational levels [13]. Therefore, the aim of the present study is to examine the correlation between the RFFT and the CogState in a population-based sample aged 18 years and older, broadly representative for the general population of the North of the Netherlands [22], while taking into account age, education level, gender, computer experience, and physical impairments. In case of high correlations, such data facilitates comparison and/or combining data of different cohort-studies worldwide. We hypothesize that the RFFT strongly (*r* > 0.50) correlates with the executive function subtest of the CogState, and weakly (*r* ≤ 0.29), with other subtests of the CogState.

## Methods

### Study design

This study is based on a sub-cohort from the LifeLines Cohort Study. LifeLines is a multi-disciplinary prospective population-based cohort study examining in a unique three-generation design the health and health-related behaviors of 167,729 persons living in the North of The Netherlands. The present study includes a consecutive series of participants aged 18 years and older who visited the LifeLines study location in Groningen, the Netherlands between October 22^nd^ and November 29^th^ 2013. During this period all participants were invited to participate in an additional visit to complete an additional cognitive examination which consists of the RFFT and the CogState battery. This additional assessment took place approximately 2 weeks after the baseline visit by trained research assistants. A total of 509 participants participated in this additional examination.

The Lifelines Cohort Study employs a broad range of investigative procedures in assessing the biomedical, socio-demographic, behavioral, physical and psychological factors which contribute to the health and disease of the general population, with a special focus on multi-morbidity and complex genetics. Baseline assessment consisted of a physical examination, cognitive functioning assessment, drawing blood samples, collecting urine samples, and self-report questionnaires regarding demographics, health status, lifestyle and psychosocial aspects. LifeLines is a facility that is open for all researchers. Information on application and data access procedure is summarized on http://www.lifelines.net/. Details of the LifeLines study design are reported elsewhere [5, 23]. Briefly, the participant recruitment and baseline assessment started in 2006 and was finished in 2013 and was performed in 12 local research sites. The LifeLines adult study population is shown to be broadly representative for the general adult population of the north of the Netherlands [22]. A three generation design and recruitment strategy was adopted to include participants [5, 23] Firstly, an index population aged 25–49 years was recruited via participating general practitioners (GPs), unless the participating GP considered the patient not eligible based on the following criteria: a) severe psychiatric or physical illness; b) limited life expectancy (<5 years); or c) insufficient knowledge of the Dutch language to complete a Dutch questionnaire. Subsequently, older and younger family members were invited by LifeLines to take part. In addition, adults could self-register to participate via the LifeLines website [5]. The participants aged between 25 and 49 years and the percentage of women are overrepresented in the LifeLines Cohort Study compared to the general population [22]. However, the mean age of the study population of the current study (mean: 53; SD: 14.6) is somewhat higher than the mean age of the study population of the LifeLines Cohort Study (mean: 45; SD: 13.1) and our study includes more males (50% versus 41) and higher educated participants (76% versus 69%). Although age distribution in the current study is not representative for the general population (i.e. there is an overrepresentation of participants aged 50 years and over) due to the recruitment strategy, for the current study it is also important to have sufficient variability in scores on cognitive functioning. All ages of 18 years and older are represented in the current study and although changes in cognitive performance can be observed in younger participants, higher variability in cognitive functioning is expected in older participants [6, 13]. Furthermore, a decline in cognitive functioning by age is also shown in higher educated participants [6]. All participants gave informed consent before they received an invitation for the physical examination. The LifeLines Cohort Study is conducted according to the principles of the Declaration of Helsinki and approved by the medical ethical committee of the University Medical Center Groningen, The Netherlands.

### Measurements

The *RFFT* consists of five parts and each part consists of 35 identical five-dot patterns arranged in seven rows and five columns on a sheet of paper. However, the stimulus pattern differs between each of the five parts. In part 1, the five-dot pattern forms a regular pentagon. Parts 2 and 3 contain the same five-dot pattern as part 1 but includes various distractors (i.e. diamonds in part 2, and lines in part 3). In parts 4 and 5 there are no distracting elements, but the five-dot pattern is a variation of the pattern of part 1 [6]. The task is to draw as many unique designs as possible within one minute by connecting the dots in different patterns. The test has been developed as a measure of nonverbal fluency and executive functioning, defined as the ability to utilize one or more strategies that maximize response production while at the same time avoiding or minimizing response repetition [7, 24]. Studies support the construct validity of the RFFT as a measure of initiation, planning and divergent reasoning. Performance on the RFFT is expressed as the total number of unique designs (the sum of all five parts, possible range: 0–175). The error ratio (i.e. the total number of perseverative errors (i.e. repetitions of designs are scored as perseverative errors) divided by the total number of unique designs [6]), is increasingly used as a measure of performance. The error ratio also reflects executive functioning, as it is an index for assessing the respondent’s ability to minimize repetition while maximizing unique productions. All participants completed the RFFT under supervision of a trained research nurse.

In the LifeLines Cohort Study, we used the CogState Brief Battery, designed to monitor cognitive change. Nonetheless, for the present study we added an executive functioning task (i.e. the Groton Maze Learning Test (GMLT)). Administration of the CogState battery was conducted on a personal computer. The total battery included the Groton Maze Learning Test (GMLT) with the delayed recall (GMLR) and the Brief Battery including four card tasks. The CogState subtasks are described in detail elsewhere [19, 25]. Briefly, instructions for each task were presented on the screen and participants were asked to carefully read these. A supervisor stayed present during the GMLT to help the participants understand the task during the practice session. During the CogState Brief Battery, no supervisor was present, although participants were informed that in case they needed assistance, a supervisor would be around to help them continue the task. The tests were administered in the following order:

1. Groton Maze Learning test (GMLT)

The GMLT is a hidden pathway maze learning task that measures executive function and spatial problem solving. This task consists of a 10 x 10 grid of tiles on a computer screen. To complete the maze, the participant must follow a hidden 28-step pathway from the start at the top left corner (indicated by a blue tile) to the finish at the bottom right of the grid (indicated by red circles). The subject is instructed to move one step from the start location and then to continue, one tile at a time, toward the end (bottom right). The participant moves by clicking a tile next to their current location using the computer mouse. After each move is made, the computer indicates whether this is correct by revealing a green checkmark, or incorrect by revealing a red cross. If a choice is incorrect (i.e. a red cross is revealed), the subject must go back to the last correct location and then make a different tile choice to advance toward the end. Once completed, participants are returned to the start location and repeat the task four more times, trying to remember the pathway they have just completed. The primary outcome measure was the total number of errors across five trails.

2. Detection task (DET)

The DET is a simple reaction time task that measures psychomotor functioning and speed of processing. In this task, the participant must attend to the center of the screen and follow the rule “Has the card turned face up? Subjects were instructed to press the “Yes” key as soon as the card turned face up. The task ended after 35 correct trials had been recorded. The primary outcome measure was reaction time (in milliseconds), which was normalized using log10 transformation.

3. Identification task (IDN)

The IDN is a choice reaction task that measures visual attention. In this task, the participant must attend to the card in the center of the screen and response to the question: “Is the card red”? Participants were required to press the “Yes” key if it is and the “No” key if it is not. This task continued until 30 correct responses have been recorded. Reaction time (in milliseconds and log10 transformed) was the primary outcome measure.

4. One Back task (OBK)

The OBK is a measure of attention and working memory. In this task, the participant must to attend to the card in the center of the screen and respond to the question “Is this card the same as that on the immediately previous trial”? If the answer was yes, participants were instructed to press the “Yes” key, and the “No” key if the answer was no. The task ends after 30 correct trials. The primary outcome measure was the proportion of correct answers, which was normalized using arcsine transformation.

5. One Card Learning task (OCL)

The OCL is a visual learning and memory task. In this task, the participant must attend to the card in the center of the screen and respond to the question “have you seen this card before in this task”? If the answer was yes, participants were instructed to press the “Yes” key, and the “No” key if the answer was no. The task ends after 42 trials. The primary outcome measure was the proportion of correct answers, normalized using arcsine transformation.

6. Groton Maze learning task – delayed recall (GMLR)

The GMLR is a measure of visual learning and memory. In this task, the 10 x 10 grid of tiles is shown again on the computer screen and participants are asked to reproduce the same hidden path as was identified in the GMLT. The participant completes this delayed recall trial once. The primary outcome measure was the total number of errors.

After the CogState battery, participants were administered a short questionnaire evaluating the CogState. Questions concerned whether participants had experience using a computer mouse (1 = never; 2 = rarely; 3 = occasionally; 4 = regularly; 5 = often), whether (physical) impairments limited them to perform the tasks (1 = yes; 2 = no), and whether participants experienced the CogState as stressful (1 = not at all stressful; 2 = a little stressful; 3 = reasonably stressful; 4 = fairly stressful; 5 = very stressful) or tiresome (1 = not at all tiresome; 2 = a little tiresome; 3 = reasonably tiresome; 4 = fairly tiresome; 5 = very tiresome).

The following participants characteristics were collected: age, gender, educational level (categorized as low (≤12 years), or high (>12 years) according to the International Standard Classification of Education (ISCED) [26]), nationality (i.e. based on the father’s and mother’s country of birth according to the definition of Statistics Netherlands [27]), marital status (being in a relationship or not), smoking status (never smoker, past smoker, or current smoker), alcohol use (no alcohol use, moderate alcohol use, or problematic alcohol use), physical activity (complying with the Dutch norm of at least half an hour of moderately intensive exercise at least 5 days a week, yes or no [28]), and the number of neurological (i.e. stroke, multiple sclerosis, epilepsy; range 0 to 3) or cardiovascular disorders (i.e. myocardial infarction, arrhythmia, heart failure, high blood pressure; range 0 to 4), diabetes (yes or no), or depression (yes or no (i.e. major or minor depression according to the Mini International Neuropsychiatric Interview (MINI) [29]).

### Statistical analysis

Sample characteristics are described by displaying percentages for categorical variables, the mean (SD) for normally distributed continuous variables and the median (IQR) for not normally distributed continuous variables.

Spearman rank correlation coefficients were calculated to compare the RFFT scores (i.e. total number of unique designs and error ratio) to the scores on the six CogState subtasks. Positive correlations are interpreted as small (*r* ≤ 0.29), medium (*r* = 0.30 to *r* = 0.49), or large (*r* ≥ 0.50) [30]. For negative correlations the same guidelines are applied for interpretation, but in opposite directions. As both cognitive scores are influenced by age, education level, and gender [6, 9, 31], we controlled for these covariates. Partial correlation could not be performed since not all assumptions were met. Therefore, we conducted subgroup analyses for: a) age (young: 18–49 years versus middle-age: 50–64 years versus older adults: ≥65 years); b) education (low versus high); and c) gender. Sensitivity analyses were performed to investigate whether having little experience using a computer mouse, being limited by (physical) impairments, or reporting one of the following conditions: problematic alcohol use, having (had) a neurological disorder (stroke, multiple sclerosis, or epilepsy), or depression, would alter the results and our conclusions, by excluding those participants from the analyses. IBM SPSS statistics software version 22 was used for the statistical analysis. Significance levels were set at *p* < 0.05 and all tests were two-tailed.

## Results

### Study sample

Of the 509 participants, 494 persons completed all six CogState subtasks and 485 persons completed the RFFT, leaving a total of 471 (93%) persons with complete data on all cognitive (sub)tasks for the correlational analyses. Table 1 shows the characteristics of the total sample and of the 471 persons for the correlation analyses separately. The mean age of the total study population at baseline was 53 years old (SD: 14.6; range: 18–87) and 50% were women. Most participants were Dutch (92%) and had a high education level (76%). The mean number of unique designs on the RFFT was 85.16 (SD: 24.37) and the median error ratio on the RFFT was 0.09 (IQR: 0.05–0.15). Scores on the CogState subtasks were measured with the GMLT (median: 52; IQR: 41–64), GMLR (median: 7; IQR: 4–10), DET (mean: 2.57; SD: 0.17), IDN (mean: 2.71; SD: 0.09), OBK (mean: 1.32; SD: 0.22), and OCL (mean: 0.97; SD: 0.13). No substantial differences were found for the total study population compared to those with complete data on all cognitive (sub)tasks.

**Table 1:** Baseline characteristics of study sample

	LifeLines sample adult population^a^		Total sample present study population		Sample present study population in the correlation analyses	
	N (%)^b^	N	N (%)^b^	N	N (%)^b^	N
Age (years), mean (SD)	45 (13.1)	152180	53 (14.6)	509	53 (14.5)	471
Gender (female)	89050 (59%)	152180	254 (50%)	509	232 (49%)	471
Education level		148679		507		469
≤ 12 years	45439 (31%)		120 (24%)		106 (23%)	
> 12 years	103240 (69%)		378 (76%)		363 (77%)	
Nationality		151575		507		469
Dutch	143040 (94%)		468 (92%)		433 (92%)	
Other	8535 (6%)		39 (8%)		36 (8%)	
Ruff Figural Fluency Test (RFFT)^c^		-				
Number of unique designs, mean (SD)			85.16 (24.37)	485	85.50 (24.18)	471
Error ratio, median (IQR)			0.09 (0.05–0.15)	485	0.09 (0.05–0.15)	471
CogState^c^		-				
Groton Maze Learning (GMLT), median (IQR)			52 (41–64)	509	52 (41–64)	471
Groton Maze Learning– Delayed Recall (GMLR), median (IQR)			7 (4–10)	504	7 (4–10)	471
Detection (DET), mean (SD)			2.57 (0.17)	500	2.56 (0.17)	471
Identification (IDN), mean (SD)			2.71 (0.09)	507	2.70 (0.08)	471
One Back (OBK), mean (SD)			1.32 (0.22)	504	1.33 (0.20)	471
One Card Learning (OCL), mean (SD)			0.97 (0.13)	506	0.98 (0.13)	471
Marital status (in a relationship)		150255		505		468
Yes	128493 (86%)		403 (80%)		374 (80%)	
No	21762 (14%)		102 (20%)		94 (20%)	
Smoking status		143245		500		462
Never smoker	68672 (48%)		214 (43%)		201 (43%)	
Past smoker	45350 (32%)		207 (41%)		189 (41%)	
Current smoker	29223 (20%)		79 (16%)		72 (16%)	
Alcohol use		136437		483		451
No alcohol use	29287 (21%)		70 (15%)		64 (14%)	
Moderate alcohol use	86613 (64%)		343 (71%)		324 (72%)	
Problematic alcohol use	20537 (15%)		70 (14%)		63 (14%)	
Physical activity^d^		138516		469		438
Yes	67240 (49%)		250 (53%)		237 (54%)	
No	71276 (51%)		219 (47%)		201 (46%)	
Number of neurological disorders^e^		150877		491		457
No disease	147564 (98%)		476 (97%)		444 (97%)	
1 disease	3228 (2%)		15 (3%)		13 (3%)	
2 diseases	85 (0.1%)		0 (0%)		0 (0%)	
Number of cardiovascular disorders^f^		111463		404		375
No disease	68921 (62%)		235 (58%)		218 (58%)	
1 disease	33510 (30%)		125 (31%)		118 (32%)	
2 diseases	8437 (8%)		40 (10%)		35 (9%)	
≥ 3 diseases	595 (1%)		4 (2%)		4 (1%)	
Depression (yes)	7538 (6%)	125987	33 (7%)	495	33 (7%)	460
Diabetes (yes)	3882 (3%)	151786	27 (5%)	507	25 (5%)	469

^a^Data available at the moment of data release of the present study; ^b^The percentage is reported, unless otherwise indicated; ^**c**^In the present study no data is available from the total LifeLines adult population; ^d^Complies with the norm of at least thirty minutes of moderately intensive exercise at least 5 days a week; ^e^Stroke, multiple sclerosis, epilepsy; ^f^Myocardial infarction, arrhythmia, heart failure, high blood pressure

In general, most participants experienced the CogState not as stressful at all (*n* = 279; 60%), or a little stressful (*n* = 178; 38%). Only few participants experienced the CogState as reasonably stressful (*n* = 7; 2%), or fairly stressful (*n* = 2; 0.4%). In addition, most participants experienced the CogState as not at all tiresome (*n* = 334; 72%), or a little tiresome (*n* = 115; 25%). Only few participants experienced the CogState as reasonably tiresome (*n* = 14; 3%), fairly tiresome (*n* = 2; 0.4%), or very tiresome (*n* = 1; 0.2%).

### Comparison of RFFT and CogState scores

Table 2 presents the results of the Spearman correlation coefficients between the scores on the RFFT and on the CogState. Scores on both RFFT outcomes (i.e. number of unique designs and error ratio) correlated statistically significant with scores on all six subtasks of the CogState, except for the correlation between the RFFT error ratio and the OBK task. Correlations were of medium strength between the RFFT number of unique designs and the DET task (*r* = -0.39) and the IDN task (*r* = -0.38). The strength of all other statistically significant correlations was small (i.e. *r* < 0.29).

**Table 2:** Spearman correlations between the RFFT and CogState (*n =* 471)^a^

	RFFT – Number of unique designs	RFFT - Error ratio	GMLT	GMLR	DET	IDN	OBK	OCL
RFFT – Number of unique designs		−0.23**	−0.28**	−0.26**	−0.39**	−0.38**	0.22**	0.21**
RFFT - Error ratio			0.28**	0.24**	0.20**	0.11**	−0.07	−0.17**
GMLT				0.73**	0.29**	0.24**	−0.16**	−0.21**
GMLR					0.26**	0.18**	−0.18**	−0.26**
DET						0.65**	−0.08	−0.14**
IDN							−0.11*	−0.13**
OBK								0.24**

*p < 0.05; **p < 0.01

^a^Including all participants aged 18 years and older with complete data on the RFFT and CogState subtasks

*RFFT* Ruff Figural Fluency Test, *GMLT* Groton Maze Learning Test, *GMLR* Groton Maze Learning Test – Delayed Recall, *DET* Detection Task, *IDN* Identification Task, *OBK*: One Back Task; *OCL*: Once Card Learning ﻿task

### Subgroup analyses

The results of the Spearman correlation coefficients between the scores on the RFFT and the CogState are presented in Tables 3, 4 and 5, separately for the following subgroups:

**Table 3:** Spearman correlations of RFFT and CogState, separate for young (18–49) (*N =* 156), middle-aged (50–64) (*N =* 226), and older (≥65) adults (*N =* 89)

	Young adults (18–49 years) (*N =* 156)	Middle-aged adults (50–64 years) (*N =* 226)	Older adults (≥65 years) (*N =* 89)
RFFT total unique designs			
GMLT	−0.15	−0.25**	−0.21*
GMLR	−0.20*	−0.21**	−0.19
DET	−0.18*	−0.30**	−0.15
IDN	−0.27**	−0.31**	−0.08
OBK	0.19*	0.08	0.43**
OCL	0.25**	0.30	0.28**
RFFT error ratio			
GMLT	0.21**	0.25**	0.23*
GMLR	0.23**	0.17*	0.26*
DET	0.11	0.05	0.15
IDN	0.01	−0.05	0.01
OBK	0.11	−0.11	−0.11
OCL	−0.20*	−0.08	−0.12

*p < 0.05; **p < 0.01; *RFFT* Ruff Figural Fluency Test, *GMLT* Groton Maze Learning Test, *GMLR* Groton Maze Learning Test – Delayed Recall, *DET* Detection Task, *IDN* Identification Task, *OBK* One Back Task' *OCL*: One Card Learning task

**Table 4:**
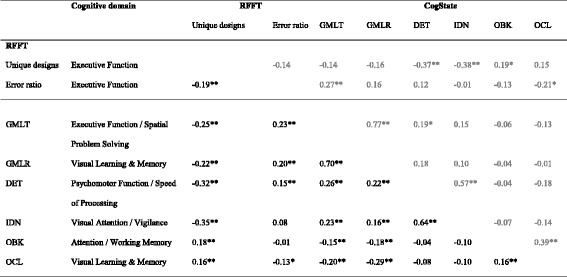
Spearman correlations of RFFT and CogState, separate for low (0–12 years) (*N =* 106) and high (>12 years) (*N =* 363) education level

*p < 0.05; **p < 0.01; *RFFT* Ruff Figural Fluency Test, *GMLT* Groton Maze Learning Test, *GMLR* Groton Maze Learning Test – Delayed Recall, *DET* Detection Task, *IDN* Identification Task, *OBK* One Back Task; *OCL*: One Card Learning task

Adults with higher education level (>12 years) are presented in black; adults with lower education level (≤12 years) are presented in grey

**Table 5:**
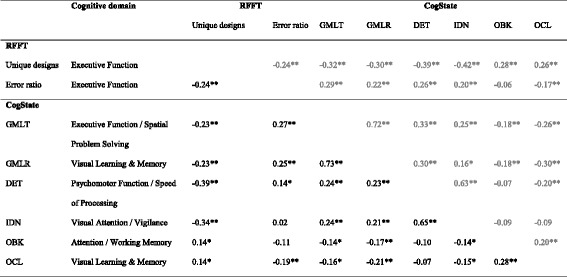
Spearman correlations of RFFT and CogState, separate for men (*N =* 239) and women (*N =* 232)

*p < 0.05; **p < 0.01; *RFFT* Ruff Figural Fluency Test, *GMLT* Groton Maze Learning Test, *GMLR* Groton Maze Learning Test – Delayed Recall, *DET* Detection Task, *IDN* Identification Task, *OBK* One Back Task; *OCL*: One Card Learning task

Females are presented in black; males are presented in grey

- *Age* (young: 18–49 years versus middle-age: 50–64 years versus older adults: ≥65 years). Among the younger participants (18–49 years, *n* = 156 (33%)), correlations between the RFFT unique designs and the CogState subtasks were comparable to the total group of participants, although generally less strong. Furthermore, the correlation between the RFFT total unique designs and the GMLT was no longer statistically significant. Among the middle-aged adults (50–64 years, *n* = 226 (48%)), correlations between the RFFT unique designs and the CogState subtasks were comparable to the total group of participants, although the correlations between the RFFT total unique designs and the OBK and the OCL were no longer statistically significant. Among the older adults (≥65 years, *n* = 89 (19%)), many correlations between the RFFT total unique designs and the CogState subtasks were no longer statistically significant. However, a correlation of medium strength was found between the RFFT number of unique designs and the OBK (*r* = 0.43) (Table 3), whereas this correlation was small (*r* = 0.22) in the total group of participants. With regard to the RFFT error ratio, all correlations were no longer significant, except for correlations between the RFFT error ratio and the GMLT and the GMLR for all age subgroups, as well as the correlation between the RFFT error ratio and the OCL for the young adult subgroup. However, the strength of these statistically significant correlations is comparable to the correlations in the total group.
- *Education (low, and high).* Among the participants with low education level (*n* = 106 (23%)), many correlations were no longer statistically significant. Among the participants with higher education levels (*n* = 363 (77%)), correlations were comparable to the total group of participants, although the correlation between the RFFT error ratio and the IDN was no longer statistically significant (Table 4).
- *Gender.* For men (*n* = 239 (51%)), a correlation of medium strength was found between the RFFT unique designs and the GMLT (*r* = -0.32) and the GMLR (*r* = -0.30) (Table 5), whereas this correlation was small in the total group of participants. Among women (*n* = 232 (49%)), correlations were comparable to the total group of participants, although the correlation between the RFFT error ratio and the IDN was no longer statistically significant.

### Sensitivity analyses

In total, 39 of 471 participants (8%) reported to never, rarely, or occasionally have used a computer mouse. These participants were slightly older than the total sample in the correlations (mean age (SD): 59 (16.2)) and included a higher percentage of lower educated persons (56%). Excluding these participants from the analyses did not change the results substantially nor did it alter our conclusions. Fourteen of 471 participants (3%) indicated that they were limited by (physical) impairments during the CogState, due to problems with their hands (*n* = 6), vision (*n* = 3), hearing (*n* = 2), or concentration (*n* = 3). Excluding those participants from the analyses did not alter the results substantially nor did it alter the conclusions, except for the correlation between the RFFT error ratio and the IDN which was no longer statistically significant (*r* =0.09; *p* > 0.05). A total of 109 of 471 participants (23%) reported a disease or addiction that might influence cognition due to problematic alcohol use (*n* = 63), having (had) a neurological disorder (*n* = 13), or having a depression (*n* = 33). Excluding those participants from the analyses did not alter the results substantially nor did it alter the conclusions, except for the correlation between the RFFT error ratio and the IDN which was no longer statistically significant (*r* =0.09; *p* > 0.05) and the correlation between the RFFT number of unique designs and GMLT which became stronger (from weak strength (*r* = -0.28; *p* < 0.01) to medium strength (*r* = -0.31; *p* < 0.01).

## Discussion

In this study, we compared cognitive functioning as measured by the RFFT to cognitive functioning measured by the CogState. We found that the RFFT significantly correlated with virtually all subtasks of the CogState, although the strength of the correlation varied. Moderate correlations were found between the RFFT number of unique designs and the DET task and the IDN task. However, the remaining correlations were weak. For future studies using cognitive measurements of the LifeLines Cohort study, this indicates that the RFFT scores measured at baseline do not translate one-to-one to CogState scores measured at follow-up. To our knowledge, this is the first study that directly compared scores of the RFFT to scores of the CogState. Other studies have compared scores of the RFFT [11, 32, 33] or the CogState [13, 18, 20] to other cognitive tests, which showed, in general, also weak to moderate, or non-significant correlations.

In our study, we would have expected a stronger correlation between the RFFT and the GMLT, as both tests are considered to measure executive functioning [6, 19]. However, executive functioning comprises a collection of higher-order cognitive processes, including planning, reasoning, working memory, inhibition, cognitive flexibility, decision-making, and self-monitoring [9, 10]. The performance of the RFFT relies on functions as initiation, planning and divergent reasoning [7, 24], but also on levels of concentration and attention, eye-hand coordination, and the use of a systematic strategy. The performance of the GMLT also relies on multiple functions in addition to executive functioning, including immediate- and short term memory for visuospatial information, and information processing speed [19]. Therefore, although both measures are considered measures of executive functioning, they do not measure exactly the same components of executive functioning. It is known that different cognitive domains are to an extend interrelated, which can be accounted for by a higher order common factor (e.g. Spearman’s General Intelligence [34]). Therefore, small to moderate correlations between different cognitive tests should be expected [35]. We chose to include the GMLT as executive functioning measurement from the CogState as we found it corresponds most with functions that are also needed to perform the RFFT. Possibly, stronger correlations could have been found between the RFFT and another executive functioning measurement from the CogState. However, we chose not to include too many tasks in our test battery, since we wanted to minimize the time needed to complete the battery, so that the participants would not get too tired or lose their concentration. Within the total sample, correlations between the RFFT number of unique designs and the DET and IDN task were the only correlations of moderate strength. Thus a second explanation, and even more likely explanation, may be that the RFFT score also reflects processing speed.

A strength of the present study is the large sample size, especially compared to previous studies on these tests. Another strength of the present study is that it includes a sample with a wide range of age and education level, resulting in a broad possible variance of scores. Since scores on the RFFT and the CogState are associated with age, education level, and gender [6, 9, 31], we investigated whether correlations between RFFT scores and CogState scores would differ between groups. The variance in scores on the cognitive tasks in our study (represented as standard deviations and interquartile ranges) was generally larger among older persons (compared to younger persons), among persons with lower education levels (compared to persons with higher education levels), and among men (compared to women). Therefore, we expected to find stronger correlations between the RFFT and the CogState among these subgroups [30]. However, our subgroup analyses for age, education level, or gender did not show substantially different results nor did it alter our conclusions. The loss of statistical significance in some subgroups (especially the older participants) is most likely explained by loss of statistical power, as the strength of the association did not change substantially. One possible explanation why our subgroup analyses for age, education level, and gender did not alter our conclusions, could lie in the study design. Participants were invited for an additional visit during which the CogState was administered. Persons with cognitive limitations are therefore less likely to participate in this study because of the extra burden of an additional visit. Moreover, previous studies showed that in general, individuals with higher age, lower socioeconomic status, with chronic diseases, or with lower levels of functioning, are less likely to participate in large population based cohort studies like LifeLines [22]. However, when comparing the scores on the RFFT and on the four CogState brief battery tasks of our study to scores from other studies including healthy controls, we find comparable scores [6, 9, 18, 25]. Furthermore, we performed sensitivity analyses to investigate the effect of computer experience on performance of the CogState. Although participants with little experience using a computer mouse were slightly older and had a lower education level compared the total sample. Sensitivity analyses in which we excluded these participants from the analyses, did not change the results substantially nor did it alter our conclusions. This is in line with studies showing that the CogState has high acceptability and efficiency and is therefore very well usable for cognitive testing among older persons, with performance levels consistent with those observed in younger groups [15].

Our study has also some limitations. Unfortunately, we were not able to investigate the criterion validity of the RFFT and the CogState (i.e. ability of these tests to detect cases of MCI or dementia in the present sample [36]), due to the lack of a gold standard (i.e. based on international diagnostic guidelines or clinical judgment following a full assessment battery) in the present study. Furthermore, the CogState Brief Battery was administered unsupervised in the present study. The advantage of administration of the CogState in a clinical setting is that a supervisor can help participants understand the task during the practice session. Participants assessed in an unsupervised research setting may be more easily distracted, show sub-optimal effort and motivation, or may have lower scores due to inadequate understanding of the task [37]. However, the CogState is increasingly administered in an unsupervised or home setting [13, 37, 38], making it less labor-intensive and less costly than a supervised clinical setting and standard pencil and paper testing. It has been shown that there were no differences in results between supervised and unsupervised settings [37]. If any, participants assessed at home performed slightly faster because they could schedule their assessment at a time they felt their freshest [13]. Therefore, it is unlikely that our unsupervised setting during the CogState brief battery influenced the results.

## Conclusions

In conclusion, our results show that cognitive functioning as measured by the RFFT does not relate one-to-one to cognitive functioning as measured by six different subtasks of the CogState. Albeit executive functioning measured with the RFFT and the GLMT were significantly correlated, the size of this correlation was only low and below our expectation (*r* > 0.50). Therefore, within the LifeLines Cohort Study, a change in cognitive functioning as measured by the RFFT at baseline cannot be deduced from CogState scores during follow-up. Nonetheless, computerized testing such as the CogState may be very well suited for large cohort studies to assess cognitive functioning in the general population and to identify cognitive changes as early as possible, as it is a less time- and labor intensive tool.
